# A childhood-onset intestinal toxemia botulism during chemotherapy for relapsed acute leukemia

**DOI:** 10.1186/s12941-017-0240-y

**Published:** 2017-09-18

**Authors:** Noriko Ohyama, Michiko Torio, Kentaro Nakashima, Yuuki Koga, Shunsuke Kanno, Hisanori Nishio, Kei Nishiyama, Momoko Sasazuki, Haru Kato, Hiroshi Asakura, Satoshi Akamine, Masafumi Sanefuji, Yoshito Ishizaki, Yasunari Sakai, Shouichi Ohga

**Affiliations:** 1 Department of Pediatrics, Graduate School of Medical Sciences, Kyushu University, 3-1-1 Maidashi, Higashi-ku, Fukuoka, 812-8582 Japan; 2 Department of Bacteriology II, National Institute of Infectious Diseases, Tokyo, Japan; 3 Division of Biomedical Food Research, National Institute of Health Sciences, Tokyo, Japan

**Keywords:** Intestinal toxemia botulism, Childhood, Chemotherapy, Antibiotics, Acute leukemia

## Abstract

**Background:**

Botulism is a potentially fatal infection characterized by progressive muscle weakness, bulbar paralysis, constipation and other autonomic dysfunctions. A recent report suggested that cancer chemotherapy might increase the risk for the intestinal toxemia botulism in both adults and children.

**Case presentation:**

We report a 5-year-old boy, who developed general muscle weakness, constipation, ptosis and mydriasis during the third induction therapy for relapsed acute myeloid leukemia. He had recent histories of multiple antibiotic therapy for bacteremia and intake of well water at home. Repeated bacterial cultures identified *Clostridium botulinum* producing botulinum neurotoxin A. Botulinum toxin A was isolated from his stools at 17, 21, and 23 days after the onset. Symptoms were self-limiting, and were fully recovered without anti-botulinum toxin globulin therapy.

**Conclusion:**

This is the second report of a pediatric case with cancer chemotherapy-associated intestinal toxemia botulism. Our case provides further evidence that the immunocompromised status due to anti-cancer treatments increases the risk for the development of botulism at all ages in childhood.

## Background

Botulism is a potentially fatal infection caused by botulinum neurotoxin (BoNT) [1, 2]. Typical symptoms include progressive paralysis with or without respiratory failure and autonomic dysfunctions. Four naturally occurring forms of botulism are known: classic food born botulism, wound botulism, infant botulism, and botulism resulting from the intestinal colonization [1]. The latter two forms of botulism are also referred to as intestinal toxemia botulism. This form of botulism rarely causes clinical signs in adults and children older than 1 year of age, while various pathological conditions that may lead to aberrant intestinal bacterial flora in intestines are known to increase the risk for its onset in both childhood and adults. Thus, particular attentions have been paid to patients at postoperative status [3], or those with Meckel’s diverticula [4], inflammatory bowel diseases [5], antimicrobial therapy [4] and bone marrow transplantation [6].

In this report, we present the case of a 5-year-old child who developed intestinal toxemia botulism during cancer chemotherapy. This is the second report of intestinal toxemia botulism associated with childhood cancer.

## Case presentation

**Fig. 1:**
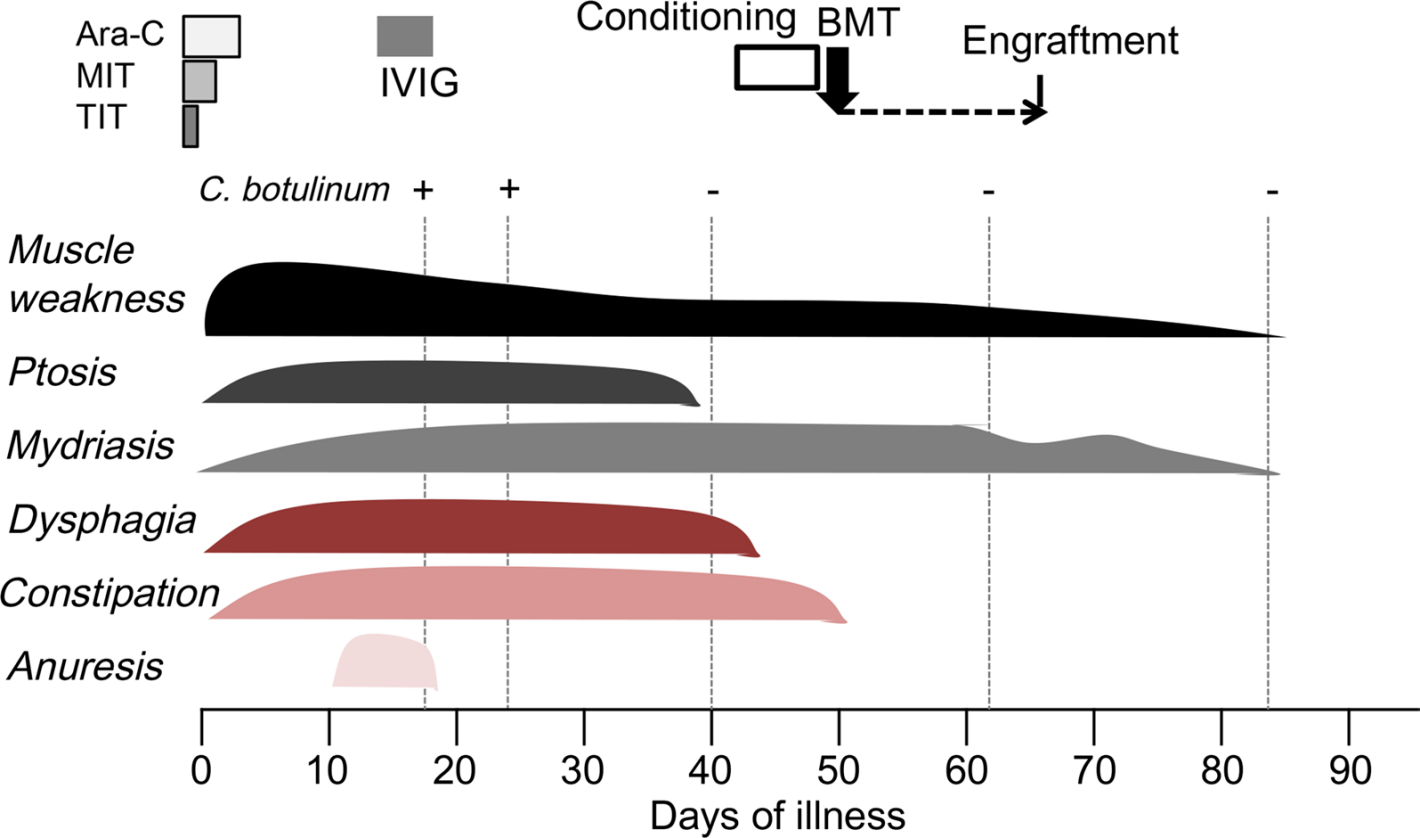
Time course of treatments and neurological symptoms. At top, treatment courses of chemotherapies (gray rectangles), conditioning therapy (white box), and bone marrow tranplantation (BMT, the black arrow) are shown. “Engraftment” was obtained at day 65. High-dose immunoglobulin therapy (IVIG, 400 mg/kg/day) was conducted from day 14 to 18 of illness. Positive (+) and negative (−) signs above vertical dash lines denote the results of tests for *C. botulinum*-culture. Shaded lobs clarify the durations and peaks of annotated neurological symptoms. *MIT* mitoxantrone, *TIT* triple intrathecal chemotherapy

The child here described was born to healthy, non consanguineous parents. His postnatal development in motor, cognitive and language skills were normal. At 3 years of age, he was diagnosed as having acute myeloid leukemia, M5. Although he obtained a complete remission after combined chemotherapy, he had a recurrence with the same type of leukemia at 5 years of age. Induction therapy was initiated with the standard regimen in Japan [7]. He received a combination of intravenous antibiotics (piperacillin, tazobactam, meropenem and teicoplanin) for the treatment of transient bacteremia with *Streptococcus* species. After the clinical resolution, he was discharged from the hospital for 13 days. On his return to our hospital, he underwent the third course of chemotherapy (Fig. 1). His mother noted bilateral ptosis, dysarthria and walking difficulty on the 2nd day of admission. Motor disability rapidly progressed. He was unable to sit up alone or roll over within a next few days. Urinary retention and constipation also developed during these days, whereas cognitive functions and sensory system were spared.

On neurological examination, general muscular hypotonia with facial weakness was prominent. Pupils were bilaterally dilated to 5 mm with sluggish light reflexes. Vertical ocular movements were restricted. Deep tendon reflexes were weakly evoked at biceps and ankles while pathological reflexes were absent. Blood tests showed unremarkable results. Anti-acetylcholine receptor and P/Q-type voltage-gated calcium channel antibodies were negative. The lumbar puncture excluded inflammatory conditions in the central nervous system. Magnetic resonance imaging studies revealed no parenchymal lesions in the brain and spinal cord. Electrophysiological tests showed marginally decreased motor nerve conduction velocity to 40.7 m/s and apparently decreased compound muscle action potential to 2.9 mV at the right median nerve. Electromyograms did not induce waxing and waning signs when stimulated at 3 and 10 Hz.

*Clostridium botulinum* was isolated from his stools at days 17, 21 and 23 of illness (Fig. 1). PCR detected both BoNT/A and B genes, and the virulence tests for mice showed the presence of BoNT/A. From these results, the diagnosis was confirmed as botulism caused by BoNT/A. Neither *C. botulinum* nor BoNT were detected from the same lot of suspected causative food product (vacuum-packaged boiled rice) that he had eaten 1 month before the onset. He was therefore diagnosed as having intestinal toxemia botulism. His parents reported that his family members have safely taken the untreated, private well water for more than 30 years. Bacterial tests of the well water were not performed because the reginal public health department considered the case as a non-familial, sporadic case of intestinal toxemia botulism. Thus, this case was not indicated for the mandated examination, and his family refused to take the voluntary survey for the contamination of botulinum spores in the well water. Therefore, the route of infection remained unknown.

His neurological symptoms began to remit from day 14 of illness without the administration of human anti-botulinum immunoglobulin. Urinary retention, constipation and dysphagia disappeared by day 40, whereas the mydriasis and muscle weakness lasted until day 90. He successfully underwent bone marrow transplantation on day 49. The neurological symptoms were not exacerbated during the conditioning therapy and after the transplantation. Complete engraftment was obtained on day 65. He was able to walk independently at the hospital discharge on day 90 of illness.

## Discussion

We presented a rare case with intestinal toxemia botulism occurred in childhood cancer treatment. The first case was reported from California, US more than a decade ago [6]. We reasoned that paucity of reported cases attribute mainly to the combination of the following host condition and environmental factors: (1) altered gut bacterial flora due to cancer chemotherapy and precedent use of antibiotics, (2) increased toxin exposure due to mucosal damage and (3) the frequent intake of contaminated foods or water.

The host immune system and the mucosal barrier against bacterial pathogens are damaged during cancer chemotherapy, and allow the increased risk for pathogenic bacterial infections [8, 9]. Cancer chemotherapy and the combined use of antibiotics can be therefore thought as a prerequisite for the outgrowth of *C. botulinum* from ingested spores, thereby leading to the onset of intestinal toxemia botulism in both adults and post-infantile children. Mucosal damage can be even greater in cases of graft-versus-host disease after bone marrow transplantation, which well explained the profound symptoms of a single reported case [6].

The progressive motor weakness and autonomic dysfunctions are the hallmark of the presentation of botulism. On the other hand, milder and transient symptoms might be undervalued as a general lethargy during the cancer chemotherapy or as its induced peripheral neuropathy. Taking these into account, we herein raise the possibility that only a portion of patients affected by intestinal toxin botulinum have been reported in the literature. Awareness of mild botulism and careful monitoring of stools may lead to the early and accurate diagnosis of intestinal toxemia botulism during cancer treatments. Through these efforts, we will further uncover the genetic susceptibility factors to the onset of this infectious disease among the general population.

## Conclusion

This report demonstrates that intestinal toxemia botulism occurs at any ages in childhood during cancer chemotherapy. Altered intestinal bacterial flora due to the chemotherapy and the combined use of antibiotics may increase the risk for the post-infantile onset of botulism. Early diagnosis after the onset of progressive paralysis is likely the key to obtain successful cure of this condition. Accumulating clinical, bacteriological and genetic data from similar cases will gain useful information on the susceptibility factors to the complication of intestinal toxemia botulism during cancer treatments.
